# The Impact of Regional Anesthesia in Masking Acute Compartment Syndrome after Limb Trauma

**DOI:** 10.3390/jcm13061787

**Published:** 2024-03-20

**Authors:** Nicole Hilber, Anna Dodi, Stephan Blumenthal, Heinz Bruppacher, Alain Borgeat, José Aguirre

**Affiliations:** 1 Institute of Anesthesiology, City Hospital Zurich, 8008 Zurich, Switzerlandstephan.blumenthal@stadtspital.ch (S.B.); josealejandro.aguirre@stadtspital.ch (J.A.); 2 Department of Anesthesiology, Balgrist Campus Zurich, 8008 Zurich, Switzerland; alain.borgeat@balgrist.ch; 3 Department of Surgery, University of Illinois at Chicago, Chicago, IL 60607, USA

**Keywords:** regional anesthesia, limb trauma, compartment syndrome, anesthesia, pain management, nerve block

## Abstract

Regional anesthesia has shown to be successful in controlling major pain in trauma patients. However, the possibility of masking acute compartment syndrome (ACS) after peripheral nerve blocks for limb injuries is still controversially discussed. Therefore, we aimed to summarize the current literature regarding this topic to shed light on the impact of peripheral regional anesthesia on the diagnosis of ACS in trauma patients. We searched Pubmed, Google Scholar and the Cochrane Library for literature following the PRISMA (preferred reporting items for systematic reviews and meta-analyses) guidelines. The analysis of these reports was included in the context of the current literature concerning this topic. We found no (randomized) studies, and only six case reports dealing with the impact of peripheral nerve blocks and ACS in patients after a limb trauma met our criteria and were included in our review. Only one reported a delay in the diagnosis of ACS. In most of the cases (5 of 6), the breakthrough pain, despite the nerve block, proved to be a good indicator of a developing ACS. However, despite some narrative articles about the topic including some recommendations about the possibly safe use of regional anesthesia techniques for limb trauma, there is still no international consensus and only one national guideline focusing on the possibly safe use of peripheral nerve blocks in trauma patients at risk of ACS. After reviewing the respective literature, we consider that intra-articular analgesia, sensory blocks, fascial plane blocks and low-concentration continuous peripheral nerve blocks are effective for analgesia and a low-risk analgesia tool for trauma and postsurgical patients at risk of ACS due to the fact that they do not lead to a dense block. Finally, we summarized suggestions based on the results of the literature for the different regional anesthesia modalities in these patients in a table to facilitate the use of these techniques.

## 1. Introduction

Peripheral (continuous) regional anesthesia is considered a highly effective analgesia regimen after elective and trauma surgery [1], avoiding the complications caused by opioids such as dizziness, nausea and vomiting and urinary retention. Moreover, its positive impact on the long-term functional outcome after elective large joint replacement has also been described [2]. However, its use is controversially discussed due to anecdotal reports [3] blaming peripheral nerve blocks for masking an incipient ACS [4]. There is limited knowledge concerning the impact of regional anesthesia on the diagnosis of ACS [4,5,6], as highlighted by the American and European Societies of regional anesthesia (ASRA, ESRA) [7]. In fact, the American College of Surgeons National Surgical Quality Improvement Project (ACS-NSQIP) published data where no differences in postoperative complications after lower extremity fractures comparing a regional or general anesthesia regimen could be shown [8]. Moreover, Zadrazil et al. recently published a case series of 565 pediatric patients using regional anesthesia for pain treatment after extremity trauma and could not describe a single case of ACS [9].

This controversy has pushed a Working Party established by the Association of Anaesthetists of Great Britain and Ireland to publish the first Guidelines dealing with regional analgesia for lower leg trauma and the risk of ACS [10]. They offered a multi-professional, consensus opinion based on an objective, narrative review of case reports and case series, aiming to provide pragmatic guidance for optimal analgesia and highlighting the need for careful observation of ACS in any patient at risk, independent of the analgesia regimen. Based on our previous publications [5,6,11], and encouraged by the topic of this special issue, we performed a systematic review, screening all studies, case reports/series and reviews available with the aim of summarizing the literature regarding the impact of peripheral regional anesthesia of the upper and lower extremities in trauma on ACS. However, to draw a complete picture about the impact of regional anesthesia and ACS, we studied the current literature dealing with other regional anesthesia modalities and summarized all results in a final table to facilitate the use of these techniques in the trauma setting.

### 1.1. What Is Acute Compartment Syndrome?

The definition of ACS includes a pressure increase within a fixed osteofascial anatomic space. The high pressure leads to decreased tissue perfusion and to an impairment of the cellular function. It can end in persistent damage with considerable functional loss after muscle necrosis [12,13]. Three factors influence the outcome in the case of increased compartment pressure: the amount and the duration of the pressure as well as the severity of the soft tissue damage. Severe injuries to soft tissues and fractures are the main causes of developing ACS [4,14]. ACS is less common in women and children than in men [6,14]. In a developing ACS, pain is considered to be a clinical symptom of pivotal importance. However, muscle tenseness, paresthesia and paresis might also indicate ACS. No palpable pulses are considered a late sign and are associated with a poor functional outcome [5,6,11]. The pain increases with stretching of the involved muscle compartment and is often reported as more intense and severe than should be expected for the injury. Moreover, pain worsens with time, and does not respond to increasing doses of pain medication, including opioids and boluses of local anesthetics applied through a peripheral nerve catheter [5,6,10,11] (Table 1). 

The data from the Royal Infirmary of Edinburgh report an annual incidence of 3.1 per 100,000 people (7.3 per 100,000 men and 0.7 per 100,000 women) on average [15]. ACS is commonly seen in males and in patients younger than 35 years [16]. After extremity trauma, 40% of all cases of ACS are described after tibial shaft fracture, whereas 23% are described after soft tissue tibial trauma and 18% after forearm fractures [17,18]. The incidence of ACS in children is lower despite the fact that they show a higher preexisting compartment pressure [19,20].

A crucial fact is that ACS might also be present in the absence of fractures. Different medical conditions with abnormal bleeding diatheses (clotting disorders, hemophilia, etc.), neurocognitive impairment and neurologic disorders with reduced sensitivity and sensibility of the limbs, as well as intramedullary nails, vascular injury, burns, high energy injury and the use of tourniquets are associated with an increased risk of ACS [15].

### 1.2. Diagnosis of Acute Compartment Syndrome

Clinical symptoms and signs are summarized in Table 1. It is important to recognize that the specificity of clinical signs is 97–98%; however, their sensitivity is as low as 13–19% [6]. In the presence of one clinical symptom, the probability of diagnosing ACS correctly is 25%. This probability increases to 93% if three clinical symptoms are present. [6].

The measurement of compartment pressure is the golden standard to determine if fasciotomy is indicated or not [11]. It is crucial that an immediate diagnosis is made, followed by surgical treatment to prevent further damage to the tissues; therefore, an objective measurement of the compartment pressure has to be performed using of the commercially available pressure devices [21]. Interestingly, there is no final consensus about the threshold value of compartmental pressure and its relation to systolic, diastolic or mean blood pressure for the diagnosis and treatment of ACS [22]. 

A noninvasive tool which might show an incipient ACS is near-infrared spectroscopy (NIRS), which analyzes the relative oxygen saturation (rScO_2_) of tissue hemoglobin [23,24].

NIRS can measure changes in local muscle oxygen saturation, offering continuous, noninvasive monitoring of intra-compartmental ischemia and hypoxia [25,26].

### 1.3. Treatment of ACS

The only treatment to avoid permanent damage after diagnosis of ACS is a surgical decompression of the affected osteofascial compartments [27,28]. The outcome after fasciotomy actually depends on the timing and the additional injuries. A delay of surgical ACS treatment for more than 12 h impairs the outcome [29,30,31]. In fact, Hayakawa et al. reported that performing a fasciotomy within 6 h after ACS diagnosis showed a satisfactory outcome in 88% of cases, leading to an amputation rate of 3.2% and a mortality rate of 2%. However, a fasciotomy performed after 12 h had a satisfactory outcome in 15% of cases, leading to 14% amputations and 4.3% reported deaths [32]. However, there are reports of residual functional impairment if fasciotomy is delayed for only 2 h after ACS diagnosis [33,34,35,36,37,38]. The surgical fasciotomy can be performed safely under general or (short-acting) regional anesthesia [21].

## 2. Methods

Pubmed, Google Scholar and the Cochrane Library were searched for literature concerning compartment syndrome in the upper and lower extremities in combination with a peripheral nerve block (PNB) and trauma in adult patients in the time period January 1980–June 2023. We excluded articles written in any languages other than English. The use of only intravenous opioid patient-controlled analgesia (PCA) and elective surgeries was also excluded. We used keywords such as ‘acute compartment syndrome’, ‘upper extremity’, ‘lower extremity’, ‘trauma’, ‘peripheral nerve block’, ‘nerve block’, ‘regional anesthesia’, ‘compartment syndrome’, ‘upper limb’ and ‘lower limb’, in different combinations. We followed the PRISMA guidelines. Some of the articles were cross-referenced. 

We also studied articles concentrating on other regional anesthesia techniques used for trauma patients, like central blocks (epidural (EDA) and spinal anesthesia), intra-articular analgesia, intravenous regional anesthesia (IVRA), wound infusion and fascial plane blocks for extremity surgery, to check the references and to summarize the data reported and offer suggestions based on the results of the literature for the different regional anesthesia modalities. 

## 3. Results

**Figure 1:**
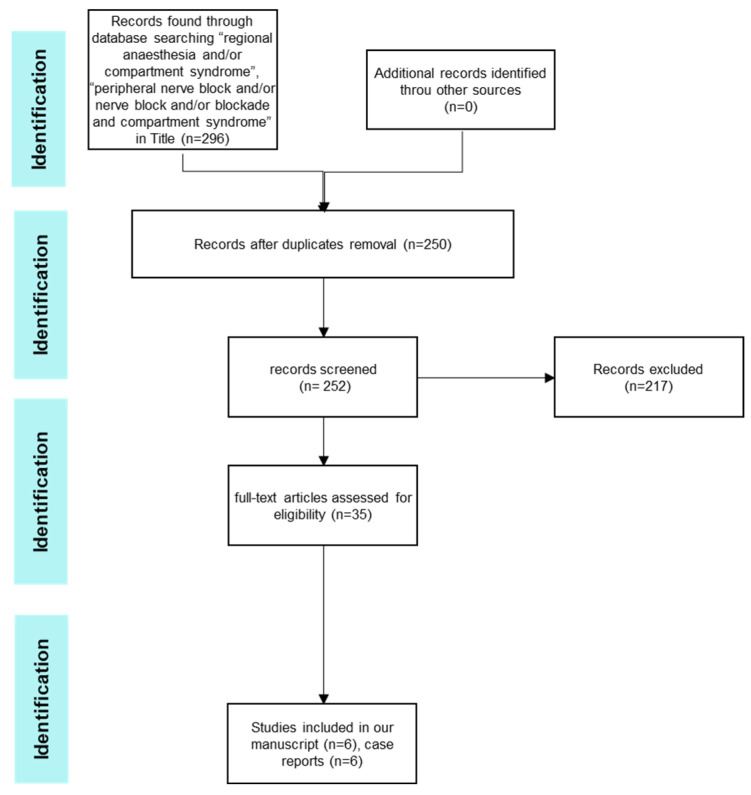
Data search diagram.

We identified 296 citations, 90 of which were related to the topic: compartment syndrome and regional anesthesia in the reviewed time period. We excluded duplicates (n = 46), articles including only PCA and no regional anesthesia (n = 8) and articles not in English (n = 3), which gave us a total of 35 full-text articles. We further excluded from the primary analysis articles including peripheral blocks for elective surgery (6), articles including patients in a study protocol for liposomal bupivacaine (1), articles including pediatric trauma and regional anesthesia (1), articles dealing with IVRA (7) and articles including spinal/EDA (12). Therefore, we included a total of six full-text articles in the final analysis: case reports (n = 6) (Table 2) (Figure 1).

Only one case reported a possible delay in the diagnosis of ACS. Ganeshan et al. [39] reported a case of a 75-year-old man with a distal radius fracture. The surgery was performed after an axillary nerve block. However, the local anesthetic used, and its concentration and volume were not reported. After the failure of K-wires, a volar periarticular locking plate was put in place. After surgery, the patient was sent home. Unfortunately, the authors did not report if the patient was discharged after the block had worn off with full sensory and motor blockade. There was no documented monitoring of the patient for signs/symptoms of developing ACS in the time frame from post-surgery until block resolution. Only 24 h after surgery did the patient present to the emergency department with blisters, and loss of sensation and motor function in the fingers and wrist. 

The case by Hyder et al. [3] was published in an orthopedic journal with the title “Compartment syndrome in tibial shaft fracture missed because of a local nerve block”, clearly suggesting that regional anesthesia was the cause of a delayed diagnosis of ACS. In this case, a 28-year-old male received an intramedullary nailing to fix a closed tibial shaft fracture. Postoperatively, the patient received a former called 3 in 1 block (a femoral nerve with uncertain spread to the obturator and lateral cutaneous nerve or the thigh) for pain control. The patient’s complaints about paresthesia of the foot were attributed to the triple nerve block. The symptoms persisted, and 48 h later, he could not flex his great toe. A fasciotomy was performed, but after surgery, the patient needed an ankle-foot orthosis for walking due to anterior tibial compartment necrosis. 

Rauf et al. [40] reported a case of a young man with a mid-shaft fracture of the radius and a malalignment after ORIF (open reduction internal fixation). The patient received a supraclavicular block using a mixture of lidocaine 2%, adrenaline and bupivacaine 0.5% prior to general anesthesia. Twenty minutes after extubation, the patient complained about dull and severe pain in his forearm. Despite the administration of paracetamol, diclofenac and morphine, the pain did not resolve and a dense sensory and motor blockade was still present. No compartment pressure monitoring was performed, but after the removal of the cast, the radial pulse was absent. The wound was explored in the operating room. A bleeding vessel was identified as causing the clinical symptoms. There was no fasciotomy performed and the patient did not suffer any long-term disabilities. The patients of the other reported cases [5,39,41,42] suffered severe pain up to a visual analogue scale (VAS) 10/10, despite a functioning peripheral nerve block. All cases using peripheral nerve blocks are summarized in Table 2.

### 3.1. Other Regional Anesthesia Techniques and ACS

#### 3.1.1. Neuraxial Blocks

We did not find a case where a single shot epidural or single shot spinal anesthesia was implicated in the delay of an ACS. However, continuous epidural analgesia has been reported to mask symptoms of ACS, delaying its diagnosis [43]. Mar and colleagues [43] reviewed 23 cases where continuous epidural analgesia (EDA) was blamed for masking ACS, and they could show that in 90% of the cases, patients showed the classical symptoms of ACS but these symptoms were not recognized in time. Breakthrough pain, a heavy clinical indicator of ACS, was ignored in most cases. However, a dense block caused by continuous EDA delayed the diagnosis of ACS in four cases.

#### 3.1.2. Intravenous Regional Anesthesia

Different reports have implicated intravenous RA (IVRA) in causing ACS [44,45,46,47,48,49,50]. 

In the cases describing ACS after IVRA, the use of prolonged and high-pressure tourniquets as well as the extravascular or erroneous injection of a foreign substance into the forearm venous system were the most likely causes of ACS.

#### 3.1.3. Single Shot/Continuous Intra-Articular Injection 

After local infiltration analgesia (LIA) or continuous wound infusion (CWI), there is no report of a delay in diagnosis of ACS.

#### 3.1.4. Compartment/Fascial Plane Blocks

After compartment/fascial plane block, there is no report of a delay in diagnosis of ACS.

**Table 2:** Cases included in this review.

Author	Gender	Age	Injury	Procedures	Nerve Block	Local Anesthetics	Symptoms of ACS	Diagnostic/ Treatment/ Time after ACS Diagnosis	Comments
Munk-Andersen [41]	Male	12 y	Distal tibia and fibula fracture	Temporary external fixation, debridement	Preop: SS distal sciatic nerve block Postop: SS distal sciatic nerve block POD 1: US-guided distal sciatic nerve catheter	Preop: Lidocaine 2%, 10 mL Postop: Ropivacaine 0.375%, 20 mL 1. postop day: Lidocaine 2% bolus, Ropivacaine 0.2% 4 mL/h	Circa 7 h after PNC, the calf muscles were tense and sore. After 8.5 h, sudden, severe pain, worsened by passive foot movement	No CP-measurement/ Fasciotomy immediate after the appearance of severe pain worsened by passive movement of the foot.	No delay due to PNB. Increased post OP myoglobine, indicated muscle ischemia. No permanent damage was reported.
Uzel [42]	Male	26 y	Third-degree, closed transverse fracture of the left femur	Splint, centromedullary nailing 15 h and 15 min after the accident. The procedure took 80 min.	SS femoral block preoperatively in combination with GA	Ropivacaine 0.75%, 20 mL	Thigh pain ca. 2 h post OP, 16 h later unusually severe pain (VAS 9/10), no sensomotor deficit, pressure in the ventral compartment 54 mmHg	No CP-measurement prior to fasciotomy. Fasciotomy of the anterior thigh compartment in SA. Apart from ventral compartment, intraoperatively, other compartments showed normal pressures.	No delay due to PNB. Severe breakthrough pain present. 16 h after FNB, probably no persistent anesthesia. No permanent damage was reported.
Ganeshan [39]	Male	75 y	Distal radius fracture	Initially K-wires placed, after 3 w, they became loose. The K-wires were removed and a plaster below elbow cast was applied. 3 w later, internal fixation with a volar peri-articular locking plate.	Axillary nerve block	Not reported	24 h after discharge, swelling of the forearm and fingers, hemorrhagic blisters, loss of sensation in the fingers, loss of active movements in the fingers and wrist. Passive movement of the fingers led to severe pain. Finger capillary refill increased to >4 s.	CP measurement: 46 mmHg and 50 mmHg in the anterior and 22 mmHg in the posterior compartment. Operation within 1 h of presentation. Fasciotomy and excision of unhealthy muscle.	LA used, neurological status at discharge, recovery time, start of symptoms after block worn off: not reported. Unlikely that an LA lasts for 24 h after an axillary block for ambulatory surgery. Discharge with risk of ACS with no telephone control of recovery and pain status to be blamed.
Aguirre [5]	Female	47 y	Complex distal humerus fracture	Open reposition, osteosynthesis of the capitulum, trochlea humeri including radial condyles and open arm splint. The procedure took 150 min.	Preop: IFC no LA administered until postoperative checking of the sensomotor function.	Initial bolus 30 mL 0.5% ropi, CI ropi 0.3% at 6 mL/h, additional bolus of 5 mL, lockout time 20 min.	Severe forearm pain (VAS 9/10) 14 h post OP. Persistent pain despite ropi 0.5% 20 mL bolus and complete motor and sensory blockade.	CP measurement of extensor compartment (40 mmHg) with fasciotomy 1 h thereafter.	No. Persistent pain despite IFC and bolus ropi. No permanent damage was reported.
Rauf [40]	Male	19 y	Mid-shaft fracture of the radius	Revision surgery because of malalignment of the radial plate 12 d earlier.	Preoperatively a SCB prior to GA.	10 mL lido 2% + adrenaline and 10 mL Bupi 0.5%	20 min after extubation, dull pain in the forearm developed during further 20 min. 2 h post block severe pain (VAS 10/10), not responsive to analgesics and despite a dense sensory and motor block. Swollen and tense forearm, no palpable radial pulse, prolonged capillary refill time after cast removal.	Immediate exploration under GA. A bleeding vessel was secured and hematoma cleared out. No fasciotomy. No wound closure, sterile occlusive dressing. 6 h after SCB, signs of block resolution. Time from clinical presentation until surgery was <30 min.	No. Breakthrough pain, which did not resolve after administration of morphine, paracetamol and diclofenac. No permanent damage was reported.
Hyder [3]	Male	28 y	Closed fracture of the tibial shaft	Intramedullary nailing, initially stabilized with a plaster cast.	After surgery: “triple nerve block” (former 3 in one block) was performed.	Bupi 0.5%	Altered sensations in his foot and leg, initially varying in areas. After 48 h, there was an inability to actively extend the big toe.	CP measurement after 48 h: 108 mmHg in the anterior compartment. Fasciotomy (timeframe unclear after diagnosis) showed dead muscles.	No. The block did not impair the sensomotor areas described (sciatic nerve). 48 h duration unlikely after Bupi. Patient walked thereafter with an orthosis.

Abbreviations: Bupi: Bupivacaine; CI: continuous infusion; CP: Compartment pressure; FNB: femoral nerve block; GA: general anesthesia; IFC: infraclavicular catheter; LA: local anesthetic; lido: lidocaine; PNB: Peripheral nerve block, PNC: peripheral nerve catheter; POD: postoperative day; Ropi: ropivacaine; SA: spinal anesthesia; SCB: supraclavicular bock; SS: single shot; VAS: visual analogue scale.

## 4. Discussion

Of the included six case reports dealing with peripheral nerve blocks and ACS, only one remains unclear. In the report by Ganeshan et al., without further information about the medications used for the blockade, and with unclear monitoring for ACS symptoms after the surgery, it is difficult to state that the peripheral nerve block delayed ACS diagnosis [39]. Additionally, ambulatory surgery in patients at risk of developing ACS remains controversial and close post-discharge monitoring is highly recommended [49]. The timeline remains unclear: when did the block wear off? When did pain start? Why did the patient wait for so long and present to the emergency department only the day after surgery with blisters, and motor and sensory compromise of the wrist and fingers? [39]. This patient suffered from persistent dysfunctions of the hand and wrist after surgery for ACS and is an unfortunate example of the need for some basic guidelines for this specific complication in the ambulatory surgery setting: not to be alone at home the first night after surgery, written information about postoperative care, a phone number of healthcare professionals as well as a follow-up call the day after surgery. Mobile apps and remote monitoring will possibly improve postoperative follow-up [51]. The case presented by Hyder et al. [3] is a clear misunderstanding of basic anatomy. After a femoral nerve block with or without the involvement of other nerves of the lumbar plexus, the sciatic nerve remains unblocked. Moreover, it would only cover analgesia in the area of the insertion of the medullar nail for an analgesia duration of approximately 8 h [11,52]. Despite this anatomical fact, and the clear misunderstanding of local anesthetic pharmacology, Tran et al. [53] recently blamed this block for the delay of ACS. Severe breakthrough pain reaching a VAS 10/10 shows that the peripheral nerve blockade did not impair or preclude ACS diagnosis. Such sudden and severe pain must make the attending doctor suspicious, and an incipient ACS has to be ruled out [5].

We are aware that there is not much literature concerning the question if a peripheral nerve block may delay the diagnosis of ACS. However, in most of the articles blaming regional anesthesia for masking ACS, there was an epidural analgesia (EDA) or an intravenous opioid patient-controlled analgesia (PCA) present. Actually, PCA and continuous EDA might mask the symptoms of acute compartment syndrome [4,16,19,20,21,22,23,24,31]. In fact, single shot epidural or single shot spinal anesthesia have not been associated with ACS [14,54,55,56,57,58,59,60,61]. 

However, continuous epidural analgesia has been reported to mask symptoms of ACS, delaying its diagnosis. In the review by Mar and colleagues [43], continuous epidural analgesia (EDA) was blamed for masking ACS in 23 cases and they could show that in 90% of the cases, patients showed the classical symptoms of ACS but these symptoms were not recognized in time. Breakthrough pain, a heavy clinical indicator of ACS, was ignored in most cases. However, a dense block caused by continuous EDA delayed the diagnosis of ACS in four cases.

In the recent Pro-Con debate about the use of PNB for trauma patients, authors highlighted the fact that a developing ACS with breakthrough pain might be more easily detected [62].

Considering other regional anesthesia techniques, intravenous RA (IVRA) has been implicated in causing ACS [44,45,46,47,48]. IVRA is frequently performed in trauma in different parts of the world and the concept of causing ACS performing an IVRA is not well understood [49,50]. The most controversial theories focus on the double tourniquet used for this technique and emphasize the ischemia-reperfusion injury leading to hyperemia, swelling and the additional application of high volumes of local anesthetics and adjuvants into a “newly created compartment.” [45]. Additionally, other factors like inflation pressure and duration as well as the use of hypertonic saline infusion [44,47,48] have also been involved in the development of ACS. According to the literature, ACS after IVRA can be caused by (1) placing the intravenous line and injection into the radial artery, (2) an idiosyncratic allergic reaction to the local anesthetic or the preservative and (3) inadvertent injection of an inappropriate agent into the forearm venous system [44,47,48]. 

Continuous wound (articular) infusion (CWI) or peri-articular infiltration (local infiltration analgesia [LIA]) are effective regional analgesia techniques but have no impact on motor or major sensory block due to the lack of effect on major nerves [63,64]. The surgery most profiting from these techniques is total knee arthroplasty, whereas hip surgery and upper extremity trauma are controversially discussed [65]. Fascial plane blocks represent a modern regional analgesia approach, where high volumes of low-concentration local anesthetics are injected for analgesia, therefore avoiding central blocks or PNBs. Fascial plane blocks lead to minimal, if at all, motor block and therefore offer an underestimated alternative for patients at risk of ACS. In fact, classic perineural blocks could be replaced by techniques like midfemoral saphenous nerve blocks for medial tibia plateau fractures, supra-inguinal fascia iliaca blocks for femur neck fractures and quadratus lumborum blocks for hip fractures [66,67]. 

The articles we included are case reports and therefore represent only class IV evidence according to the Agency for Healthcare Research and Quality (AHRQ). This is the main limitation of this review. There are no randomized controlled studies available on this topic. There are some articles stating that regional anesthesia does not delay the diagnosis of ACS [28,29], but these studies are also based on case series, which limits the evidence. Considering the fact that even PCA has been blamed for masking ACS [16,20,21,22], using well-adapted regional anesthesia techniques might offer different advantages. Apart from the avoidance of opioid-related side effects, the breakthrough pain, through a functioning continuous peripheral nerve block, can be used as an early diagnostic tool, as shown in five of our included cases. In fact, breakthrough pain persisted after systemic analgesics and even after a top up of the perineural catheter. As stated by Aguirre et al., ‘proper documentation and a high level of suspicion coupled with postoperative repeated clinical and, if needed, invasive monitoring are of utmost importance’ [3]. 

## 5. Conclusions

Due to the low quality of data, only six case reports, it is difficult to state that regional anesthesia could routinely be used in trauma patients. Five of these case reports show that regional anesthesia did not mask the diagnosis of ACS and the sixth case report remains inconclusive due to missing data. We can state that if surgeons and anesthesiologists keep a high index of suspicion, adapt the regional anesthesia techniques and use basic clinical monitoring in patients at risk of ACS, regional anesthesia remains a valuable option for good postoperative pain management. Even though in most case reports high concentrations of local anesthetics were used when breakthrough pain was present, we recommend the use of local anesthetics, volumes and concentrations to avoid dense and long-lasting blocks. Moreover, ACS must be excluded when (breakthrough) pain cannot be managed despite a well-placed continuous regional anesthesia. Due to the unclear literature concerning IVRA and ACS, this technique should not be used in trauma surgery [3,25]. Moreover, dense motor blocks [3,32] and high-concentration epidural catheters [43] should be avoided; we recommend lower concentrations and higher flowrates to achieve that. 

We summarized the Recommendations from the ESRA/ASRA Joint Committee Statement [7] and the Recommendations of the Association of Anaesthetists of Great Britain and Ireland [10] (Table 3 and Table 4) to display the two existing Anesthesia Societies’ recommendations on this topic. Moreover, based on the literature reported in this review and our experience [5], we summarized possible suggestions for the different regional anesthesia modalities to apply in patients at risk of ACS in a table to facilitate the use of these techniques (Table 5).

## Figures and Tables

**Table 1:** Symptoms and signs of acute compartment syndrome.

Symptoms	Signs
Pain is greater than expected or increasing Paresthesia in affected extremity Splinting or removal of casts leads to no relief Raise in pain and analgesic demand	Pain after passive stretching of the respective compartment Swollen and tense compartment Pallor Pulselessness (late sign) Muscle weakness Sensory deficit of the nerves enclosed in the compartment
Note: In the early stage of ACS, pulses might be present, but they are absent in the late stage. Therefore, palpable pulses do not exclude ACS. During the early development of ACS, the capillary refill is present. ACS can occur in open fractures. Clinical signs remain unclear due to their low specificity and sensitivity. After regional anesthesia or opioid patient-controlled analgesia (PCA), probably more sensitive clinical signs of ACS: - Breakthrough pain despite well-working regional anesthesia. - Increase demand of analgesics.	

**Table 3:** Recommendations from the ESRA/ASRA Joint Committee Statement, modified according to Ivani G et al. [7].

All patients with regional anaesthesia/acute pain should be followed by the actue pain service. Perform compartment pressure measurement if ACS is suspected.
If regional anesthesia is performed in patients at high risk of ACS, the dose (volume and concentration) of local anesthetics should be reduced.
Carefully evaluate the use of adjuvants due to the possible increase in block duration and block intensity. Use bupivacaine, levobupivacaine or ropivacaine at concentraions of 0.1–0.25% for single shot peripheral nerve blocks and neuraxial blocks.
Use bupivacaine 0.125% or ropivacaine 0.1–0.2% at rates of 0.1–0.3 mg/kg/hr for continuous peripheral nerve blocks and continuous neuraxial blocks.

**Table 4:** Recommendations of the Association of Anaesthetists of Great Britain and Ireland, modified according to Nathanson NH et al. [10].

Manage patients at risk of ACS within agreed, multidisciplinary protocols.
Trained staff should be able to indentify signs and symptoms of ACS in the postoperative period. The use of objective scoring charts is recommended.
All surgery or trauma patients should be offered effective analgesia after full explanation and documented informed consent.
In the case of no consensus between anesthetist and surgeon, the role of the anaesthetist as the expert on pain relief should be respected.
Avoid the use of neuraxial or peripheral regional techniques resulting in dense blocks of long duration significantly exceeding the duration of surgery.
Use lower concentrations of local anaesthetic drugs without adjuncts for single shot or continuous peripheral nerve blocks provided post-injury, and postoperative surveillance is appropriate and effective to avoid delays in diagnosis of ACS. Due to the lack of reliable, published data on the safety and efficacy of analgesia in patients at risk of ACS and as prospective randomized trials would need to be large due to the low evidence of ACS, the Working Party recommends the conduct of prospective audits.

**Table 5:** Suggestions for anesthesia and postoperative analgesia in patients at high-risk of postoperative ACS.

Anesthesia Techniques	Drugs to Be Used	Duration of Action	Recommendation for Trauma
Single shot PNB (SPNB)	Lidocaine 1.5% Mepivacaine 1% Chloroprocaine 2–3%	Lidocaine: 2.5–3 h Mepivacaine: 2–4 h Chloroprocaine: 1–2 h	For low postoperative pain, adapted local anesthetics to surgery time. Consider low-dose CPNB.
Continuous PNB (CPNB)	Ropivacaine: bolus with 10–20 mL of 0.1–0.2% PCRA: ropivacaine 0.1–0.2% (0.3%) 4–6 mL/h, bolus 3–4 mL, lock out 20–30 min	While infused and 30–60 min after stopping the infusion. No motor function impairment at low dosages.	Consider if catheter placement possible without previous block (or the block is performed with short-acting LA or low-concentration LA to avoid a long-lasting, dense block)
CWI/IAI/(C)FPB	Ropivacaine 0.2–0.3% Bupivacaine 0.25%. Dexamethasone I.V. 8–12 mg for FPB	Covers pain only during infusion.	Use whenever possible: good analgesia, no case report blaming this technique for masking ACS.
Single shot spinal (SSPA)	Bupivacaine 0.5% hyperbaric/isobaric low-dose (7.5 mg–max 10 mg); if needed add fentanyl/clonidine Mepivacaine 1% (30 mg) Chloroprocaine 1% 50 mg Prilocaine 2% hyper/isobaric 30–60 mg	Bupivacaine: 3–4 h Mepivacaine: 2–3 h Chloroprocaine: 1–2 h Prilocaine: 1.5–2.5 h	Use for lower limb trauma if possible to adapt duration to surgery time. No case report blaming SSPA for masking ACS.
Continuous spinal (CSPA)	Surgery: Bupivacaine (isobaric or) hyperbaric 0.5% during surgery 0.5–2 mL initial bolus, thereafter adaptation to surgery time and sensory level. Analgesia: Bupivacaine isobaric 0.125–0.2% for 0.5–1 mL/h	Bupivacaine: 2–3.5 h	No published case blaming CSPA for masking ACS. However, dense, long-lasting motor block possible if used also after surgery. Therefore, use CSPA for longer lasting surgery and in cases GA is not the optimal choice. Avoid using CSPA for analgesia after surgery if risk of ACS due to possible dense block.
Single shot epidural (EDA)	Lidocaine 1.5% Chloroporcaine 3% (Ropivacaine 0.75%–1%)	Lidocaine: 3.5 h Chloroprocaine: 2.5 h Ropivacaine: 3–6 h	No published case blaming EDA for masking ACS. However, a dense motor block is possible. Use EDA in cases GA is not the optimal choice.
Continuous epidural (CEDA)	Ropivacaine 0.1% (−0.2%) Levobupivacaine 0.125%; if needed add sufentanil 1 µ/mL, fentanyl 1–3 µ/mL	While infused and 2–4 h after stopping the infusion. A block resolution within 60 min achieved after wash out with 30 mL saline.	Avoid if GA, SPA or CPNB possible. Different case reports blaming CEDA for masking ACS.
General anesthesia (GA)	Propofol/volatile anesthetics Low-dose long-acting opioids (fentanyl); remifentanil TCI until low-concentration CPNB start possible.	Remifentanil: 5 min after TCI is stopped.	Avoid ideally for high-risk patients. If GA, combine with CPNB for postoperative analgesia.

Abbreviations: ACS: Acute compartment syndrome, (c) FPB: (continuous) fascia plane block, CWI: continuous wound infusion, EDA: epidural anesthesia, IAI: intra-articular infusion, LA: local anesthetic, PNB: peripheral nerve block, SPA: spinal anesthesia.

## References

[1] Gadsden J., Warlick A. (2015). Regional anesthesia for the trauma patient: Improving patient outcomes. Local Reg. Anesth..

[2] Atchabahian A., Schwartz G., Hall C.B., Lajam C.M., Andreae M.H. (2015). Regional analgesia for improvement of long-term functional outcome after elective large joint replacement. Cochrane Database Syst. Rev..

[3] Hyder N., Kessler S., Jennings A.G., De Boer P.G. (1996). Compartment syndrome in tibial shaft fracture missed because of a local nerve block. J. Bone Jt. Surg. Br..

[4] Marhofer P., Halm J., Feigl G.C., Schepers T., Hollmann M.W. (2021). Regional Anesthesia and Compartment Syndrome. Anesth. Analg..

[5] Aguirre J.A., Gresch D., Popovici A., Bernhard J., Borgeat A. (2013). Case scenario: Compartment syndrome of the forearm in patient with an infraclavicular catheter: Breakthrough pain as indicator. Anesthesiology.

[6] Aguirre J.A., Wolmarans M., Borgeat A. (2022). Acute Extremity Compartment Syndrome and (Regional): Anesthesia: The Monster Under the Bed. Anesthesiol. Clin..

[7] Ivani G., Suresh S., Ecoffey C., Bosenberg A., Lonnqvist P.A., Krane E., Veyckemans F., Polaner D.M., Van de Velde M., Neal J.M. (2015). The European Society of Regional Anaesthesia and Pain Therapy and the American Society of Regional Anesthesia and Pain Medicine Joint Committee Practice Advisory on Controversial Topics in Pediatric Regional Anesthesia. Reg. Anesth. Pain Med..

[8] Brovman E.Y., Wallace F.C., Weaver M.J., Beutler S.S., Urman R.D. (2019). Anesthesia Type Is Not Associated with Postoperative Complications in the Care of Patients with Lower Extremity Traumatic Fractures. Anesth. Analg..

[9] Zadrazil M., Opfermann P., Marhofer P., Westerlund A.I., Haider T. (2020). Brachial plexus block with ultrasound guidance for upper-limb trauma surgery in children: A retrospective cohort study of 565 cases. Br. J. Anaesth..

[10] Nathanson M.H., Harrop-Griffiths W., Aldington D.J., Forward D., Mannion S., Kinnear-Mellor R.G.M., Miller K.L., Ratnayake B., Wiles M.D., Wolmarans M.R. (2021). Regional analgesia for lower leg trauma and the risk of acute compartment syndrome: Guideline from the Association of Anaesthetists. Anaesthesia.

[11] Aguirre J., De Andrés J. (2018). The role of regional anestheisa (RA) in patients at risk for acute compartment syndrome (ACS). Update on Regional Anesthesia and Pain Management.

[12] Friedrich J.B., Shin A.Y. (2007). Management of forearm compartment syndrome. Hand Clin..

[13] Sigamoney K., Khincha P., Badge R., Shah N. (2015). Compartment sydrome: Challenges and solutions. Orthop. Res. Rev..

[14] Dunwoody J.M., Reichert C.C., Brown K.L. (1997). Compartment syndrome associated with bupivacaine and fentanyl epidural analgesia in pediatric orthopaedics. J. Pediatr. Orthop..

[15] McQueen M.M., Gaston P., Court-Brown C.M. (2000). Acute compartment syndrome. Who is at risk?. J. Bone Jt. Surg. Br..

[16] Martin J.T. (1992). Compartment syndromes: Concepts and perspectives for the anesthesiologist. Anesth. Analg..

[17] Noorpuri B.S., Shahane S.A., Getty C.J. (2000). Acute compartment syndrome following revisional arthroplasty of the forefoot: The dangers of ankle-block. Foot Ankle Int..

[18] Elliott K.G., Johnstone A.J. (2003). Diagnosing acute compartment syndrome. J. Bone Jt. Surg. Br..

[19] Cometa M.A., Esch A.T., Boezaart A.P. (2011). Did continuous femoral and sciatic nerve block obscure the diagnosis or delay the treatment of acute lower leg compartment syndrome? A case report. Pain Med..

[20] Klucka J., Stourac P., Stouracova A., Masek M., Repko M. (2017). Compartment syndrome and regional anaesthesia: Critical review. Biomed Pap. Med. Fac. Univ. Palacky Olomouc Czech Repub..

[21] Murdock M., Murdoch M.M. (2012). Compartment syndrome: A review of the literature. Clin. Podiatr. Med. Surg..

[22] Janzing H.M. (2007). Epidemiology, Etiology, Pathophysiology and Diagnosis of the Acute Compartment Syndrome of the Extremity. Eur. J. Trauma Emerg. Surg..

[23] Arbabi S., Brundage S.I., Gentilello L.M. (1999). Near-infrared spectroscopy: A potential method for continuous, transcutaneous monitoring for compartmental syndrome in critically injured patients. J. Trauma.

[24] Shuler M.S., Reisman W.M., Kinsey T.L., Whitesides T.E., Hammerberg E.M., Davila M.G., Moore T.J. (2010). Correlation between muscle oxygenation and compartment pressures in acute compartment syndrome of the leg. J. Bone Jt. Surg. Am..

[25] Rolfe P. (2000). In vivo near-infrared spectroscopy. Annu. Rev. Biomed. Eng..

[26] Widder S., Ranson M.K., Zygun D., Knox L., Laupland K.B., Laird P., Ball C.G., Kirkpatrick A.W. (2008). Use of near-infrared spectroscopy as a physiologic monitor for intra-abdominal hypertension. J. Trauma.

[27] Shadgan B., Menon M., Sanders D., Berry G., Martin C., Duffy P., Stephen D., O’Brien P.J. (2010). Current thinking about acute compartment syndrome of the lower extremity. Can. J. Surg.

[28] Kashuk J.L., Moore E.E., Pinski S., Johnson J.L., Moore J.B., Morgan S., Cothren C.C., Smith W. (2009). Lower extremity compartment syndrome in the acute care surgery paradigm: Safety lessons learned. Patient Saf. Surg..

[29] Heckman M.M., Whitesides T.E., Grewe S.R., Rooks M.D. (1994). Compartment pressure in association with closed tibial fractures. The relationship between tissue pressure, compartment, and the distance from the site of the fracture. J. Bone Jt. Surg. Am..

[30] Geary N. (1984). Late surgical decompression for compartment syndrome of the forearm. J. Bone Jt. Surg. Br..

[31] Janzing H., Broos P., Rommens P. (1996). Compartment syndrome as a complication of skin traction in children with femoral fractures. J. Trauma.

[32] Hayakawa H., Aldington D.J., Moore R.A. (2009). Acute traumatic compartment syndrome: A systematic review of results of fasciotomy. Trauma.

[33] Cascio B.M., Pateder D.B., Wilckens J.H., Frassica F.J. (2005). Compartment syndrome: Time from diagnosis to fasciotomy. J. Surg. Orthop. Adv..

[34] Walker B.J., Noonan K.J., Bosenberg A.T. (2012). Evolving compartment syndrome not masked by a continuous peripheral nerve block: Evidence-based case management. Reg. Anesth. Pain Med..

[35] Kucera T.J., Boezaart A.P. (2014). Regional anesthesia does not consistently block ischemic pain: Two further cases and a review of the literature. Pain Med..

[36] Sermeus L., Boeckx S., Camerlynck H., Somville J., Vercauteren M. (2015). Postsurgical compartment syndrome of the forearm diagnosed in a child receiving a continuous infra-clavicular peripheral nerve block. Acta Anaesthesiol. Belg..

[37] Torrie A., Sharma J., Mason M., Cruz Eng H. (2017). Regional anesthesia did not delay diagnosis of compartment syndrome: A case report of anterior compartment syndrome in the thigh not masked by an adductor canal catheter. Am. J. Case Rep..

[38] Camelo C.R., Eklund S.E., Brusseau R., Gomez-Morad A.D. (2020). Ischemic pain not masked by regional anesthesia. Reg. Anesth. Pain Med..

[39] Ganeshan R.M., Mamoowala N., Ward M., Sochart D. (2015). Acute compartment syndrome risk in fracture fixation with regional blocks. BMJ Case Rep..

[40] Rauf J., Iohom G., O’Donnell B. (2015). Acute compartment syndrome and regional anaesthesia—A case report. Rom. J. Anaesth. Intensive Care.

[41] Munk-Andersen H., Laustrup T.K. (2013). Compartment syndrome diagnosed in due time by breakthrough pain despite continuous peripheral nerve block. Acta Anaesthesiol. Scand..

[42] Uzel A.P., Steinmann G. (2009). Thigh compartment syndrome after intramedullary femoral nailing: Possible femoral nerve block influence on diagnosis timing. Orthop. Traumatol. Surg. Res..

[43] Mar G.J., Barrington M.J., McGuirk B.R. (2009). Acute compartment syndrome of the lower limb and the effect of postoperative analgesia on diagnosis. Br. J. Anaesth..

[44] Guay J. (2009). Adverse events associated with intravenous regional anesthesia (Bier block): A systematic review of complications. J. Clin. Anesth..

[45] Ananthanarayan C., Castro C., McKee N., Sakotic G. (2000). Compartment syndrome following intravenous regional anesthesia. Can. J. Anaesth..

[46] Maletis G.B., Watson R.C., Scott S. (1989). Compartment syndrome. A complication of intravenous regional anesthesia in the reduction of lower leg shaft fractures. Orthopedics.

[47] Hastings H., Misamore G. (1987). Compartment syndrome resulting from intravenous regional anesthesia. J. Hand Surg. Am..

[48] Mabee J.R., Bostwick T.L., Burke M.K. (1994). Iatrogenic compartment syndrome from hypertonic saline injection in Bier block. J. Emerg. Med..

[49] Luce E.A., Mangubat E. (1983). Loss of hand and forearm following Bier block: A case report. J. Hand Surg. Am..

[50] Ball C.M. (1985). Failed intravenous arm block or diagnostic test?. Anaesth. Intensive Care..

[51] Rohi A., Olofsson M.E.T., Jakobsson J.G. (2022). Ambulatory anesthesia and discharge: An update around guidelines and trends. Curr. Opin. Anaesthesiol..

[52] Eyres K.S., Hill G., Magides A. (1996). Compartment syndrome in tibial shaft fracture missed because of a local nerve block. J. Bone Jt. Surg. Br..

[53] Tran A.A., Lee D., Fassihi S.C., Smith E., Lee R., Siram G. (2020). A systematic review of the effect of regional anesthesia on diagnosis and management of acute compartment syndrome in long bone fractures. Eur. J. Trauma Emerg. Surg..

[54] Beerle B.J., Rose R.J. (1993). Lower extremity compartment syndrome from prolonged lithotomy position not masked by epidural bupivacaine and fentanyl. Reg. Anesth..

[55] Goldsmith A.L., McCallum M.I. (1996). Compartment syndrome as a complication of the prolonged use of the Lloyd-Davies position. Anaesthesia.

[56] Heyn J., Ladurner R., Ozimek A., Vogel T., Hallfeldt K.K., Mussack T. (2006). Gluteal compartment syndrome after prostatectomy caused by incorrect positioning. Eur. J. Med. Res..

[57] Iwasaka H., Itoh K., Miyakawa H., Noguchi T., Kitano T., Taniguchi K., Honda N. (1993). Compartment syndrome after prolonged lithotomy position in patient receiving combined epidural and general anesthesia. J. Anesth..

[58] Llewellyn N., Moriarty A. (2007). The national pediatric epidural audit. Paediatr Anaesth..

[59] Montgomery C.J., Ready L.B. (1991). Epidural opioid analgesia does not obscure diagnosis of compartment syndrome resulting from prolonged lithotomy position. Anesthesiology.

[60] Stotts A.K., Carroll K.L., Schafer P.G., Santora S.D., Branigan T.D. (2003). Medial compartment syndrome of the foot: An unusual complication of spine surgery. Spine.

[61] Tuckey J. (1996). Bilateral compartment syndrome complicating prolonged lithotomy position. Br. J. Anaesth..

[62] Samet R.E., Torrie A.M., Chembrovich S.V., Ihnatsenka B.V. (2023). Pro-Con Debate: Peripheral Nerve Blockade Should Be Provided Routinely in Extremity Trauma, Including in Patients at Risk for Acute Compartment Syndrome. Anesth. Analg..

[63] Liu S.S., Richman J.M., Thirlby R.C., Wu C.L. (2006). Efficacy of continuous wound catheters delivering local anesthetic for postoperative analgesia: A quantitative and qualitative systematic review of randomized controlled trials. J. Am. Coll. Surg..

[64] Aguirre J., Baulig B., Dora C., Ekatodramis G., Votta-Velis G., Ruland P., Borgeat A. (2012). Continuous epicapsular ropivacaine 0.3% infusion after minimally invasive hip arthroplasty: A prospective, randomized, double-blinded, placebo-controlled study comparing continuous wound infusion with morphine patient-controlled analgesia. Anesth. Analg..

[65] Yung E.M., Got T.C., Patel N., Brull R., Abdallah F.W. (2021). Intra-articular infiltration analgesia for arthroscopic shoulder surgery: A systematic review and meta-analysis. Anaesthesia.

[66] Chin K.J., Lirk P., Hollmann M.W., Schwarz S.K.W. (2021). Mechanisms of action of fascial plane blocks: A narrative review. Reg. Anesth. Pain Med..

[67] Black N.D., Stecco C., Chan V.W.S. (2021). Fascial Plane Blocks: More Questions Than Answers?. Anesth. Analg..

